# Auxiliary diagnostic potential of ventricle geometry and late gadolinium enhancement in left ventricular non-compaction; non-randomized case control study

**DOI:** 10.1186/s12872-017-0721-0

**Published:** 2017-12-06

**Authors:** Marko Boban, Vladimir Pesa, Ivo Darko Gabric, Sime Manola, Viktor Persic, Helena Antic-Kauzlaric, Marinko Zulj, Aleksandar Vcev

**Affiliations:** 1 Department of Cardiology, University hospital “Thalassotherapia Opatija”, Faculty of Medicine, University of Rijeka, M. Tita 188/1, 51 440 Opatija, Croatia; 2 Department of Internal medicine, Faculty of Medicine, “J.J. Strossmayer” University of Osijek, Osijek, Croatia; 3 Department of Cardiology, University Hospital “Sestre Milosrdnice” Zagreb, Zagreb, Croatia

**Keywords:** Left ventricle non-compaction (LVNC), Late gadolinium enhancement (LGE), Cardiac magnetic resonance imaging (CMR), Left ventricular geometry

## Abstract

**Background:**

There are still ambiguities existing in regard to left ventricular non-compaction (LVNC) diagnostic imaging. The aim of our study was to analyze diagnostic potential of late gadolinium enhancement (LGE) and ventricle geometry in patients with LVNC and controls.

**Methods:**

Data on cardiac magnetic resonance imaging (CMR) studies for LVNC were reassessed from the hospital’s database (3.75 years; *n*=1975 exams). Matching sample of controls included cases with no structural heart disease, hypertrophic or dilative cardiomyopathy, arrhythmogenic right ventricular dysplasia or subacute myocarditis. Eccentricity of the left ventricle was measured at end diastole in the region with pronounced NC and maximal to minimal ratio (MaxMinEDDR) was calculated.

**Results:**

Study included 255 patients referred for CMR, 100 (39.2%) with LVNC (prevalence in the studied period 5.01%) and 155 (60.8%) controls. Existing LGE had sensitivity of 52.5% (95%-CI:42.3–62.5), specificity of 80.4% (95%-CI:73.2–86.5) for LVNC, area under curve (AUC) 0.664 (95%-CI:0.603–0.722);*p*<0.001. MaxMinEDDR>1.10 had sensitivity of 95.0% (95%-CI:88.7–98.4), specificity of 82.6% (95%-CI: 75.7–88.2) for LVNC, AUC 0.917 (95%-CI:0.876–0.948); *p*<0.001. LGE correlated with Max-Min-EDD-R (Rho=0.130; *p*=0.038) and there was significant difference in ROC analysis ΔAUC0.244 (95%-CI:0.175–0.314); *p*<0.001. LGE also correlated negatively with stroke volume and systolic function (both *p*<0.05, respectively).

**Conclusions:**

LGE was found to be frequently expressed in patients with LVNC, but without sufficient power to be used as a discriminative diagnostic parameter. Both LGE and eccentricity of the left ventricle were found to be relatively solid diagnostic landmarks of complex infrastructural and functional changes within the failing heart.

## Background

Left ventricle non-compaction (LVNC) is cardiomyopathy of infrequent prevalence, but associated with serious adverse prognostic outcomes [1, 2]. There is a complex pathophysiologic background made up of inherited and external factors, whose interplay leads to alterations in cellular organization or signaling, myocardial wall maturation, loss of systolic function, increased likelihood of arrhythmias and intracardial thrombosis [3–5]. On the other side, due to great variability of clinical expression and presentation, currently available diagnostic criteria are not perfect [6]. Trabeculations of the left ventricle can indeed be found in non-negligible number of imaging studies, but their clinical significance is often inconclusive, particularly in cases in which prognostic assessment is solely based on imaging. In some cases genetic testing can offer valuable landmark that can reveal a concealed risk of sudden cardiac death [7, 8].

Specific characteristics of LVNC, such as compact (C) myocardial wall thinning, pronounced meshwork of non-compact (NC) myocardium with 2.0–2.3 ratio of NC/C and a proof of blood flow through the trabecula, taken cumulatively together make that only some cases with trabeculated myocardium fit within the LVNC cardiomyopathy [9–11]. Proportion of non-compact myocardium in the total mass of the left ventricle was also found to be a highly sensitive and specific diagnostic parameter in cases in which over one fifth of the ventricle is affected [12]. Remarkable advancements in diagnosing LVNC came with wider availability of cardiac magnetic resonance imaging (CMR) [13, 14]. Thanks to excellent insight in volumetrics, three dimensional multiplanarity, geometry and tissue changes, CMR offers valuable data for verification of the LVNC diagnosis, as well as functional analysis and insight in the prognostic course, superior to the conventional imaging modalities [12, 15]. Tissue characterization, especially the late gadolinium enhancement (LGE), offer further information on pathophysiology, differential diagnosis, as well as pathoanatomic background for increased prevalence of arrhythmias or thromboses [16, 17].

Previous studies have shown that existence of LGE in LVNC is associated with deprived prognostic course [18, 19]. Left ventricle (LV) geometry was only scarcely studied in regard to LVNC [17]. Our preliminary findings, based on large volume of exams, found that eccentricity of the left ventricle is connected with LVNC. The aim of our study was to assess the existence, characteristics and diagnostic potential of LGE in patients with LVNC and controls, using CMR. In addition, connections existing between LGE and geometry of the left ventricle were systematically assessed, with particular interest on their head to head diagnostic utility.

## Methods

Patients referred for CMR imaging of non-ischemic cardiomyopathies were prospectively included in our registry after signing an informed consent, prior to imaging procedure. Study was conducted in accordance with the Declaration of Helsinki and good clinical practice principles. Approval was obtained from the ethical board of the Hospital. There was no funding, compensations or other sources of financing, and there was no relation with medical industry. There were no additional or indirect reimbursements for patients or study personnel.

Patients included in this study were retrospectively recruited from a digital CMR data base (including analyses for diagnostic reports and image files), based on LVNC diagnosis, for a period of 3.75 years. Entire set of imaging studies was reanalyzed by two experienced cardiologists and a radiologist. LVNC diagnosis included the following CMR diagnostic criteria: non-compact-to-compact layer >2.3:1, thinning of the compact myocardium layer and trabeculations affecting >20% of the left ventricle [3, 10, 12]. There were 2 (0.1%) cases with clinically confirmed LVNC not fulfilling the criterion of NC > 20% LV. In those cases, LVNC diagnosis was confirmed with the following clinical criteria: electrocardiographic abnormalities, electrophysiology testing and in one case, family history of cardiomyopathy and sudden cardiac death. Cases with existing trabeculations whose range did not fulfill the previously mentioned CMR criteria in a clearly defined manner were included as a sub-group of controls i.e. as the *cases with suspected LVNC*. This sub-group of controls included patients with NC/C ratio range of 1.8–2.2:1 and trabeculations affecting >10% to <17% of LV. *Control group of patients* included statistically comparable sample of individuals with: no evidence of structural heart disease (healthy athletes, those with arrhythmias and those referred to CMR for ruling out of structural heart disease, prior to electrophysiology studies, cardiac thrombosis or cardiomyopathy), hypertensive heart disease (HHD), hypertrophic cardiomyopathy (HCM), dilative cardiomyopathy (DCM) (considering exclusion of significant coronary artery disease), arrhythmogenic right ventricle cardiomyopathy (ARVD) and subacute or late phase myocarditis (inclusion of outpatients). *Study did not include* patients with congenital heart disease, previous heart surgery, cardiac tumors, significant pericardial effusion, primary valvular disorder (aortic stenosis, mitral stenosis) and those with coronary artery disease (having significant coronary artery disease (known stenoses >30%), positive adenosine stress test for screening of coronary artery disease, or ischemic type of late gadolinium enhancement).

Imaging studies were performed on 1.5 T Magnetom Avanto, Siemens® (Erlangen, Germany, EU), using ECG gating and breath-hold following two respiratory cycles, Body Matrix chest and spine coils. CMR protocol consisted of setting localizers, Half-Fourier Acquisition Single-shot Turbo spin Echo (HASTE) sequences, steady state free precession (SSFP) of standard heart 2-, 4- and 3-chamber planes and 6 mm stack of short axial slices (8–12 slices through ventricle), with adding of the right ventricle and its outflow tract in case of any clinical questions. In certain cases, that was followed by short tau inversion recovery (STIR) or turbo spin echo (TSE) T1 and T2 sequences dark blood, and fat saturation sequences, which was indicated prior to or during the exam, on a case-to-case basis. Gadolinium contrast was applied in a 0.2 mL/kg (0.1 mmol/kg) dose. An intravenous bolus of Omniscan® [Gadodiamide, GE Healthcare, Little Chalfont, UK, EU] or Dotarem® [gadoterate meglumine, Guerbet, Roissy, France, EU] was used in the exams, as well as late gadolinium enhancement (LGE) sequences 20–30 min following the contrast application.

Postprocessing was performed on Siemens AG- NUMARIS/4, Syngo MR B17® software package (Erlangen, Germany, EU), whilst volumetric analyses were done using Siemens AG- Syngo Console Argus®, by a team made of two CMR high throughput cardiologists (300–450 exams per year) and a radiologist. Standard reports included a clinical questionnaire from the referring cardiologist, medical history, interpretation of all planes, sequences, tissue sequences, volumetrics, dedicated measurements of myocardial thickness in trabecula and solid part, indirect analysis of valvular function, late gadolinium enhancement and conclusions arrived at based on the exam. Final interpretation of the results was done in accordance with the standardized myocardial segmentation, as recommended by professional associations. For the purpose of this study, geometry included eccentricity analyses in the dedicated region of the left ventricle, affected by non-compaction, and the same i.e. comparable region in the control group of patients, developed by the principal author of the study (MB). Slices from the mid to apical part (chosen from the 4th - 8th layer of short axis stack of 6 mm slices, with typical range 8–12 per patient) of the left ventricle short axis single slice, created parallel to mitral valve by calculating the ratio of the longest end diastolic diameter (EDD) divided by the diameter of perpendicular line (solid to solid myocardium), i.e. maximal/minimal end diastolic ratio (MaxMinEDDR).

Population and studied groups were analyzed using descriptive statistic and presented as means combined with standard deviations or numbers with percentages. Numeric variables were analyzed for differences by the Mann-Whitney U test or Kruskal Wallis. Group data analyses were calculated with Chi square or Kruskal Wallis. Connections between the studied CMR parameters and LVNC were described by Spearman Rho. Bi-nominal regression analysis was used for the studied CMR parameters and LGE existence. LVNC diagnostic value of the late gadolinium enhancement and left ventricular geometry was first calculated separately and then in head to head settings using receiver operating characteristic (ROC) curve analysis and NC/C plus NC > 20% LV as previously standardized parameters. *P* value less than 0.05 was considered significant. Statistical analyses were done by an experienced statistician using IBM-SPSS12® v 20 (IBM co, Chicago, IL, USA) MedCalc v. 12.2® for Windows (MedCalc software co, Belgium, EU) and Statistica 10® for Windows (StatSoft inc, Tulsa, OK, USA).

## Results

### Studied sample

The study included 255 cases referred for CMR a 3.75-year period, covering 1975 exams. Control group of patients (*n* = 155; 60.8%) included cases with no structural heart disease and those with non-ischemic cardiomyopathy other than LVNC. Patients with LVNC that fulfilled all three CMR criteria (thinning, ratio and share, as previously explained) were included, making sample of one hundred (39.2%) consecutive patients (5.1% prevalence of total exams in the studied period) From those patients, only 2 cases were confirmed by addition of clinical criteria (not having >20% of trabeculations in the left ventricular mass despite having NC/C > 2.3:1). Fifty-six cases (36.1%) from the control group had clinically suspicious, but not clearly defined LVNC. Detailed data on patients and studied groups are presented in Table 1.

**Table 1:** Differences between LVNC patients and controls

	Controls	LVNC	Chi square
	*n* = 155	*n* = 100	
Male	95 (61.3%)	56 (56.0%)	0.401
Female	60 (38.7%)	44 (44.0%)	
LVEF < 50%	37 (23.9%)	48 (48.0%)	**<0.001**
OK	79 (51.0%)	0 (0.0%)	**<0.001**
LVNC	0 (0.0%)	100 (100.0%)	
DCM	27 (17.4%)	0 (0.0%)	
HCM	33 (21.3%)	0 (0.0%)	
ARVD	5 (3.2%)	0 (0.0%)	
HHD	6 (3.9%)	0 (0.0%)	
MCD	5 (3.2%)	0 (0.0%)	
	Controls	LVNC	Mann-Whitney
	*n* = 155	*n* = 100	
Age (years)	48.2 ± 15.4	45.5 ± 17.8	0.180
LA (cm2)	26.9 ± 8.6	25.6 ± 6.4	0.333
RA (cm2)	24.6 ± 7.6	23.1 ± 6.2	0.155
LVEDD (cm)	5.4 ± 1.0	5.7 ± 0.8	**<0.001**
IVS (cm)	1.2 ± 0.4	1.0 ± 0.2	**0.001**
EDV (mL)	160.3 ± 78.7	169.4 ± 61.3	**0.036**
ESV (mL)	79.4 ± 76.4	95.9 ± 69.1	**<0.001**
SV (mL)	84.4 ± 45.7	78.5 ± 25.6	0.244
LVEF (%)	55.3 ± 15.2	48.2 ± 12.7	**<0.001**
MM (gram)	122.2 ± 48.5	106.0 ± 36.3	**0.006**

*LVNC* Left ventricular non compaction, *OK* No structural heart disease, *DCM* Dilative cardiomyopathy, *HCM* Hypertophic cardiomyopathy, *ARVD* Arrhythmogenic right ventricle disease, *HHD* Hypertensive heart disease, *MCD* Myocarditis, *LVEDD (cm)* Left ventricle end diastolic dimension in 4-chamber view, *IVS (cm)* Interventricular septum thickness in 4-chamber view, *LA (cm2)* Left atrial area in square centimeters in 4-chamber view, *LVEF (%)* Left ventricle ejection fraction, *EDV (mL)* End diastolic volume, *ESV (mL)* End systolic volume, *SV (mL)* Stroke volume, *LVEF (%)* Left ventricle ejection fraction, *MM (gram)* Myocardial mass in end-diastole. Data shown as numbers with percentages or means with standard deviations (SD). Significant values are outlined in bold

### Late gadolinium enhancement

LGE was found in 133 patients (52.2%) as follows: focal mid-ventricular (MV) type was found in 35 (26.3%) cases, linear MV in 83 (62.4%) and diffuse LGE in 15 (11.3%) cases. The most common type of LGE in LVNC was linear MV, located in basal (*n* = 44) and basal/middle sections (*n* = 8) of the left ventricle, whilst the patients with no structural heart disease most commonly had no LGE imbibition.

Correlations between LGE, geometry and LVNC diagnostic parameters for the studied sample of cases are presented in Table 2.

**Table 2:** Correlations of LGE, geometry and other diagnostic parameters in the studied population sample (*n* = 255)

		LVNC	NC/C > 2.3:1	NC > 20%LV	LGE	LVEF < 50%	C	NC	NC/C	MIN EDD	MAX EDD	MaxMin EDDR
LVNC	Rho CC	NA	**0.888**	**0.960**	**0.140**	**0.234**	**−0.668**	**0.560**	**0.795**	**−0.079**	**0.234**	**0.689**
	p		**<0.001**	**<0.001**	**0.025**	**<0.001**	**<0.001**	**<0.001**	**<0.001**	**0.206**	**<0.001**	**<0.001**
MNC	Rho CC	0.032	**0.240**	NA	0.066	0.114	**−0.258**	**0.496**	**0.575**	0.032	0.061	0.093
	p	0.690	**0.003**		0.414	0.156	**0.001**	**<0.001**	**<0.001**	0.693	0.454	0.249
Age (years)	Rho CC	−0.094	−0.038	−0.095	**0.137**	0.110	0.118	0.061	−0.053	0.115	0.100	−0.087
	p	0.136	0.544	0.132	**0.029**	0.080	0.060	0.333	0.395	0.066	0.110	0.168
LA (cm2)	Rho CC	−0.063	−0.087	−0.060	**0.143**	**0.275**	**0.210**	0.075	−0.085	**0.522**	**0.506**	−0.070
	p	0.314	0.165	0.338	**0.023**	**<0.001**	**0.001**	0.233	0.176	**<0.001**	**<0.001**	0.268
EDV (mL)	Rho CC	**0.128**	0.074	**0.130**	**0.173**	**0.426**	0.058	**0.188**	0.082	**0.729**	**0.750**	0.008
	p	**0.042**	0.238	**0.039**	**0.006**	**<0.001**	0.359	**0.003**	0.192	**<0.001**	**<0.001**	0.905
ESV (mL)	Rho CC	**0.255**	**0.210**	**0.265**	**0.224**	**0.666**	−0.041	**0.294**	**0.218**	**0.685**	**0.726**	0.059
	p	**<0.001**	**0.001**	**<0.001**	**<0.001**	**<0.001**	0.512	**<0.001**	**<0.001**	**<0.001**	**<0.001**	0.344
LVEDD (cm)	Rho CC	**0.209**	**0.182**	**0.226**	**0.206**	**0.520**	0.006	**0.318**	**0.196**	**0.656**	**0.689**	0.043
	p	**0.001**	**0.004**	**<0.001**	**0.001**	**<0.001**	0.930	**<0.001**	**0.002**	**<0.001**	**<0.001**	0.491
IVS (cm)	Rho CC	**−0.196**	**−0.169**	**−0.220**	**0.262**	0.057	**0.377**	−0.026	**−0.251**	**0.202**	**0.130**	**−0.137**
	p	**0.002**	**0.007**	**<0.001**	**<0.001**	0.369	**<0.001**	0.676	**<0.001**	**0.001**	**0.037**	**0.029**
LVEF	Rho CC	**−0.313**	**−0.309**	**−0.332**	**−0.301**	**−0.809**	**0.154**	**−0.330**	**−0.311**	**−0.507**	**−0.570**	−0.117
	p	**<0.001**	**<0.001**	**<0.001**	**<0.001**	**<0.001**	**0.014**	**<0.001**	**<0.001**	**<0.001**	**<0.001**	0.062
MM (gram)	Rho CC	**−0.157**	**−0.190**	**−0.168**	**0.202**	**0.253**	**0.395**	0.025	**−0.222**	**0.554**	**0.498**	**−0.144**
	p	**0.012**	**0.002**	**0.007**	**0.001**	**<0.001**	**<0.001**	0.687	**<0.001**	**<0.001**	**<0.001**	**0.021**

*LVNC* Left ventricular non compaction, *NC/C > 2.3:1* NC to C ratio > 2.3:1 - non-compact to compact layers thickness ratio, *LGE* Late gadolinium enhancement, *NC > 20%LV* Non-compact myocardium proportion > 20% of the left ventricle, *EDD* End diastolic dimension, *Max* maximal, *Min* minimal, *MaxMinEDDR* ratio of maximal to minimal EDD, *MNC* Marginal non-compaction (clinical suspicions of LVNC), *NA* Not applicable, *LVEDD (cm)* Left ventricle end diastolic dimension in 4-chamber view, *IVS (cm)* Interventricular septum thickness in 4-chamber view, *LA (cm2)* Left atrial area in square centimeters in 4-chamber view, *LVEF (%)* Left ventricle ejection fraction, *EDV (mL)* End diastolic volume, *ESV (mL)* End systolic volume, *LVEF (%)* Left ventricle ejection fraction, *MM (gram)* Myocardial mass in end-diastole. Significant correlations presented in bold

Existence of LGE was found in cases with LVEF ≤49.2% using receiver operating characteristic curve analysis, with sensitivity of 50.8% (95%-CI:41.6–59.2, specificity of 85.1% (95%-CI:77.5–90.9), +likelihood ratio (+LR) of 3.4 (95%-CI:2.8–4.1), −LR of 0.6 (0.4–0.9), area under curve (AUC) of 0.674 (95%-CI: 0.612–0.731, *p* < 0.001.

Using binomial regression analyzes, existence of LGE was significantly connected with following studied parameters: LV-myocardial mass = 1.01 (95%-CI: 1.00–1.02), *p* = 0.048; LVEF = 0.97 (95%-CI: 0.95–0.98), *p* = 0.002 and Max-Min-EDDR 1.01 (95%-CI: 1.00–28.99), *p* = 0.045, while the NC/C was not significant.

Differences between existence of LGE in patients with LVNC and controls, with further sub-analyzes of type of LGE are presented in Tables 3 and 4.

**Table 3:** Principal characteristics and differences in the studied sample, depending on the existence and type of late gadolinium enhancement

	No LGE	LGE	Chi square	No LGE	Focal MV	Linear MV	Diffuse	Kruskal Wallis
	n (%)	n (%)		n (%)	n (%)	n (%)	n (%)	
Male	64 (52.5%)	87 (65.4%)	**0.031**	64 (52.5%)	27 (77.1%)	49 (59.0%)	11 (73.3%)	**0.038**
Female	58 (47.5%)	46 (34.6%)		58 (47.5%)	8 (22.9%)	34 (41.0%)	4 (26.7%)	
OK	67 (54.9%)	12 (9.0%)	**<0.001**	67 (54.9%)	3 (8.6%)	7 (8.4%)	2 (13.3%)	**<0.001**
LVNC	40 (32.8%)	60 (45.1%)		40 (32.8%)	4 (11.4%)	52 (62.7%)	4 (26.7%)	
DCM	4 (3.3%)	23 (17.3%)		4 (3.3%)	3 (8.6%)	18 (21.7%)	2 (13.3%)	
HCM	2 (1.6%)	31 (23.3%)		2 (1.6%)	22 (62.9%)	3 (3.6%)	6 (40.0%)	
ARVD	4 (3.3%)	1 (0.8%)		4 (3.3%)	0 (0.0%)	1 (1.2%)	0 (0.0%)	
HHD	4 (3.3%)	2 (1.5%)		4 (3.3%)	0 (0.0%)	2 (2.4%)	0 (0.0%)	
MCD	1 (0.8%)	4 (3.0%)		1 (0.8%)	3 (8.6%)	0 (0.0%)	1 (6.7%)	
LVEF < 50%	20 (16.4%)	65 (48.9%)	**<0.001**	20 (16.4%)	13 (37.1%)	48 (57.8%)	4 (26.7%)	**<0.001**
NC:C > 2.3:1	40 (32.8%)	67 (50.4%)	**0.005**	40 (32.8%)	6 (17.1%)	56 (67.5%)	5 (33.3%)	**<0.001**
LVNC	40 (32.8%)	60 (45.1%)	**<0.001**	40 (32.8%)	4 (11.4%)	52 (63.9%)	4 (26.7%)	**<0.001**
MNC	27 (32.9%)	29 (39.7%)	0.410	27 (32.9%)	10 (32.3%)	14 (45.2%)	5 (45.5%)	0.560
MCE	26 (21.3%)	59 (44.4%)	**<0.001**	26 (21.3%)	17 (48.6%)	33 (39.8%)	9 (60.0%)	**0.001**
NC > 20%LV	39 (32.0%)	59 (44.4%)	**0.047**	39 (32.0%)	3 (8.6%)	52 (62.7%)	4 (26.7%)	**<0.001**

*LGE* late gadolinium enhancement, *MV* Midventricular, *LVNC* Left ventricular non compaction, *OK* No structural heart disease, *DCM* Dilative cardiomyopathy, *HCM* Hypertophic cardiomyopathy, *ARVD* Arrhythmogenic right ventricle disease, *HHD* Hypertensive heart disease, *MCD* Myocarditis, *LVEF (%)* Left ventricle ejection fraction, *NC to C ratio* Non-compact to compact layers thickness ratio, *MNC* Marginal non-compaction (clinical suspicion of LVNC), *MCE* Multiple cardiomyopathy elements, *NC > 20%LV* Non-compact myocardium proportion > 20% of the left ventricle. Significant values outlined in bold

**Table 4:** Characteristics of myocardial layers and left ventricle geometry in dependence of the existence of late gadolinium enhancement

	No LGE	LGE	Mann-Whitney	No LGE	Focal MV	Linear MV	Diffuse	Kruskal Wallis
	Mean ± SD	Mean ± SD		Mean ± SD	Mean ± SD	Mean ± SD	Mean ± SD	
Age (years)	44.9 ± 15.3	49.1 ± 17.2	**0.029**	44.9 ± 15.3	50.8 ± 16.8	49.0 ± 17.7	46.1 ± 16.3	**<0.001**
LVEDD (cm)	5.31 ± 0.66	5.73 ± 1.06	**0.001**	5.31 ± 0.66	5.39 ± 0.83	5.95 ± 1.09	5.33 ± 1.09	0.124
IVS (cm)	0.98 ± 0.17	1.19 ± 0.43	**<0.001**	0.98 ± 0.17	1.50 ± 0.55	1.04 ± 0.22	1.35 ± 0.57	**<0.001**
RV (cm)	3.71 ± 0.68	3.58 ± 0.79	0.230	3.71 ± 0.68	3.68 ± 0.69	3.57 ± 0.84	3.43 ± 0.70	0.424
LA (cm2)	24.88 ± 5.89	27.89 ± 8.95	**0.023**	24.88 ± 5.89	28.11 ± 7.21	28.06 ± 9.78	26.40 ± 8.10	0.105
RA (cm2)	22.82 ± 6.21	25.14 ± 7.68	**0.016**	22.82 ± 6.21	25.09 ± 6.57	25.23 ± 7.83	24.80 ± 9.57	0.064
EDV (mL)	149.4 ± 45.4	177.5 ± 88.5	**0.006**	149.4 ± 45.4	156.9 ± 50.3	190.4 ± 103.2	153.1 ± 47.8	**0.014**
ESV (mL)	66.4 ± 32.5	103.9 ± 94.2	**<0.001**	66.4 ± 32.5	79.4 ± 52.3	120.8 ± 110.0	67.3 ± 39.1	**<0.001**
SV (mL)	88.1 ± 49.6	76.6 ± 25.4	**0.008**	88.1 ± 49.6	77.0 ± 21.6	74.7 ± 26.6	85.8 ± 26.0	**0.020**
LVEF (%)	57.1 ± 8.8	48.2 ± 17.4	**<0.001**	57.1 ± 8.8	53.3 ± 17.5	44.4 ± 16.6	57.6 ± 16.0	**<0.001**
MM (gram)	105.8 ± 32.7	125.2 ± 51.9	**0.001**	105.8 ± 32.7	139.2 ± 38.0	119.5 ± 57.3	125.2 ± 44.1	**<0.001**
C- thickness (cm)	0.65 ± 0.20	0.67 ± 0.24	0.853	0.65 ± 0.20	0.84 ± 0.24	0.59 ± 0.21	0.73 ± 0.23	**<0.001**
NC- thickness (cm)	1.10 ± 0.46	1.18 ± 0.48	**0.025**	1.10 ± 0.46	0.96 ± 0.51	1.31 ± 0.42	1.01 ± 0.53	**<0.001**
NC to C ratio	1.99 ± 1.29	2.14 ± 1.20	0.127	1.99 ± 1.29	1.30 ± 0.85	2.58 ± 1.09	1.65 ± 1.27	**<0.001**
Minimal MA EDD (cm)	5.16 ± 0.66	5.43 ± 1.02	**0.040**	5.16 ± 0.66	5.19 ± 0.83	5.56 ± 1.14	5.25 ± 0.39	0.130
Maximal MA EDD (cm)	5.63 ± 0.75	6.04 ± 1.06	**<0.001**	5.63 ± 0.75	5.68 ± 0.69	6.24 ± 1.21	5.77 ± 0.48	**<0.001**
Max to Min EDD Ratio	1.09 ± 0.08	1.12 ± 0.09	**0.039**	1.09 ± 0.08	1.10 ± 0.11	1.13 ± 0.08	1.10 ± 0.11	**0.005**
LV trabeculations %	14.7 ± 11.2	17.4 ± 12.2	**0.117**	14.7 ± 11.2	8.2 ± 7.7	21.7 ± 11.7	13.4 ± 10.8	**<0.001**

*LGE* Late gadolinium enhancement, *MV* Midventricular, *LVNC* Left ventricular non compaction; LV-NC % - left ventricle non-compaction proportion in total mass of the left ventricle, *C-Compact (cm)* Thickness of solid myocardium, *NC-non-compact (cm)* Thickness of trabeculated part i.e. non-compaction myocardium, *LVEDD (cm)* Left ventricle end diastolic dimension in 4-chamber view, *IVS (cm)* Interventricular septum thickness in 4-chamber view, *RV (cm)* Right ventricle end diastolic dimension in 4-chamber view, *LA & RA (cm2)* Left and right atrial area in square centimeters in 4-chamber view, *LVEF (%)* Left ventricle ejection fraction, *EDV (mL)* End diastolic volume, *ESV (mL)* End systolic volume, *SV (mL)* Stroke volume, *MM (gram)* Myocardial mass in end-diastole, *MA* Mid to apical slices in 6-mm pile stack of short axes, *EDD* End diastolic dimension, *Max* Maximal, *Min* Minimal. Data shown as mean with standard deviations (SD). Statistically significant values (*p* < 0.05) presented in bold

### Geometry of the left ventricle

**Fig. 1:**
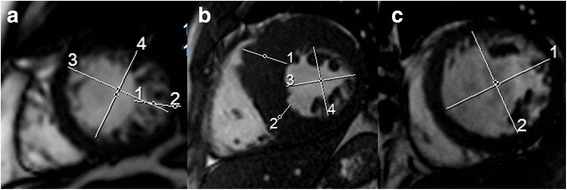
Geometry of the left ventricle in various heart diseases Data labels: **a** Case of patient with left ventricular non-compaction and trabeculations existing on 35.3% of the left ventricle, ejection fraction of 52% and confirmed non–compaction; short axis cine at end diastole (ED), marked white line showing: 1) NC-layer thickness 2.22 cm, 2) C-layer thickness 0.57 cm, with NC/C = 3.9. 3) Maximal ED dimension 6.40 cm, 4) Minimal EDD 5.59 cm, giving MaxMinEDDR = 1.14; (**b**) patient with hypertrophic cardiomyopathy: 1) 3.41 cm, 2) 2.40 cm, 3) 5.17 cm, 4) 5.16 cm i.e. Max-Min EDDR = 1.00; (**c**) patient with dilative cardiomyopathy: 1) 7.20 cm, 2) 6.99 cm i.e. Max-Min EDDR = 1.03

Max-Min-EDDR was 1.11 ± 0.09 (95%-CI: 1.01–1.26), with significant difference between the patients with LVNC and controls, 1.17 ± 0.09 vs. 1.06 ± 0.05, *p* < 0.001. Similarly, Max-Min-EDDR of LVNC was significantly different among all the sub-studied control groups (no structural heart disease, 1.06 ± 0.04; hypertensive heart disease, 1.07 ± 0.03; dilative cardiomyopathy 1.05 ± 0.04; hypertrophic cardiomyopathy 1.08 ± 0.08; ARVD 1.07 ± 0.03 and myocarditis 1.05 ± 0.01), *p* < 0.001. In addition, Max-Min-EDDR was significantly different between the patients with no LGE imbibition and controls, 1.09 ± 0.08 vs. 1.12 ± 0.09, respectively, *p* = 0.039. Figure 1. shows examples of geometric eccentricity in a patient with LVNC and two other cardiomyopathies. Correlations of Max-Min-EDDR and studied diagnostic parameters is presented in Table 2.

Model of receiver operating characteristic curve analysis of Max-Min-EDDR for detection of systolic function impairment (defined with LVEF < 50%) was not significant (AUC = 0.559; *p* = 0.121).

### Diagnostic utility of CMR parameters for LVNC

Receiver operating characteristic (ROC) curve analysis was used for diagnostic utility of the studied CMR parameters for establishing a LVNC diagnosis, in which NC/C > 2.3:1 and NC > 20%LV were considered as the gold standard.

Existing LGE had sensitivity of 52.5% (95%-CI:42.3–62.5), specificity of 80.4% (95%-CI:73.2–86.5), +LR of 2.7 (95%-CI:2.2–3.3), −LR of 0.6 (0.4–0.9), area under curve (AUC) of 0.664 (95%-CI: 0.603–0.722); *p* < 0.001.

MaxMinEDDR being >1.10 (i.e. exactly 1.0975) had sensitivity of 95.0% (95%-CI:88.7–98.4), specificity of 82.6% (95%-CI:75.7–88.2), +LR of 5.0 (95%-CI:5.0–5.9), −LR of 0.1 (0.02–0.2), area under curve (AUC) of 0.917 (95%-CI: 0.876–0.948); *p* < 0.001.

**Fig. 2:**
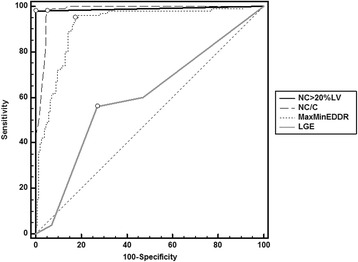
Comparison of receiver operating characteristic (ROC) curve analysis for LVNC diagnostic parameters. Data labels: LGE - late gadolinium enhancement; NC/C - non-compact to compact layer thickness ratio; NC% - non-compact myocardium percentage in total left ventricle mass; MaxMinEDDR - maximal to minimal end diastolic diameter (EDD) ratio; C - compact layer thickness; NC - non-compact layer thickness; ^◯^ - showing value with the highest Youden index

Comparison of ROC curves for the studied diagnostic parameters of LVNC was as follows: ROC-AUC (NC > 20%LV) = 0.985 (95%-CI: 0.961–0.996), ROC-AUC (NC/C > 2.3:1) = 0.983 (95%-CI: 0.959–0.995), ROC-AUC (MaxMinEDDR) = 0.909 (95%-CI: 0.866–0.941) and ROC-AUC (linear-MV-LGE) = 0.664 (95%-CI:0.603–0.722). There was also a significant difference between ROC AUC for LGE and eccentricity, ΔAUC0.244 (95%-CI:0.175–0.314), *p* < 0.001, as shown in Fig. 2.

## Discussion

This study analyzed the existence and morphology of LGE imbibitions, their connections with left ventricle geometry, as well as their diagnostic utility for LVNC. The “*gold standard*” for LVNC diagnosis using CMR was defined by thinning of compact LV wall, NC/C > 2.3:1 and NC > 20%LV [11]. In these terms, our study used substantially different methodology from several recently published registry based studies that found higher prevalence of trabeculations, but without serious prognostic implications, most likely due to not having a primary objective of LVNC diagnostics per se, different inclusion criteria and not assessing the existence of LGE [20]. In addition, impairment of systolic function of the left ventricle in our study was also more congruent with previous reports [21].

Nearly a half of the studied patients with LVNC had LGE imbibition, where the latter attained specificity and sensitivity of 55–60% as a diagnostic parameter. Proportion of patients with LGE in our study was in line with previous reports on the left ventricle non-compaction cardiomyopathy [22]. The most common pattern of LGE in LVNC was linear midventricular, followed by none, while only a small number of cases had diffuse imbibitions [22]. Interestingly, linear midventricular LGE in patients with non-compaction was located in basal and basal to middle sections of the left ventricle, whilst mid apical regions with the most pronounced trabeculations did not show clinically significant proportion of late imbibitions. Linear midventricular type of LGE was the most common LGE type in patients with dilative cardiomyopathy, focal type LGE was the most prevalent in patients with hypertrophic cardiomyopathy, while the patients with no structural heart disease predominantly had no late imbibitions [23]. There were no significant differences in LGE imbibition between the cases with suspected LVNC and the remaining part of the control group. The latter reveled the shortcomings of late gadolinium imbibition as a diagnostic tool for differentiating between the cases with increased trabeculations of the left ventricle suspected of LVNC. However, it is worthwhile to note that the difference of LGE imbibition was significant to cases with overt LVNC [24]. Existence of LGE correlated only to a mild degree with the age of patients, and there was a strong inverse correlation with systolic function of the left ventricle [25]. The former seems to represent a time-dependent degenerative process, and support the fact of overall underprivileged prognostic course in patients with heart failure [26]. Critical cut-off point to the left ventricle ejection fraction for existence of LGE was found to be set at ≤49.2%, as an intermediate-grade sensitive and high-grade specific sign of systolic impairment [24]. Existence of LGE imbibition in the left ventricle was connected with increased end diastolic dimension, interventricular septum thickness and enlarged atrial volumes, which could imply further diagnostic-prognostic value of this parameter [27]. Regarding the LV-volumetrics, LGE was in mild correlation with end diastolic volume, end systolic volume and LV-myocardial mass. Changes in cardiomyocytes, which made them susceptible for late gadolinium imbibition, consequentially increased size of the left ventricle and atria, while systolic function decreased, representing the point of down turning of the Frank-Starling curve and value of LGE as the initial diagnostic landmark of the failing heart [28, 29].

Cases with LVNC displayed structural rearrangements in terms of geometric eccentricity of mid to apical short axis sections, affected with non-compaction [30]. Eccentricity of the left ventricle was in significant and powerful correlation with LVNC diagnosis and its diagnostic parameters. MaxMinEDDR did not correlate in cases with clinical suspicion of LVNC or those with multiple cardiomyopathy elements, underscoring further diagnostic value of geometric changes for LVNC. In addition, correlation of Max-Min-EDDR with LVNC diagnostic criteria attained powerful synergy, while its constituents, taken individually had only mild or insignificant correlations. Difference between maximal and minimal end diastolic ratio of over 10% had 95% sensitivity and 83% specificity for LVNC, making it a very promising innovative diagnostic parameter. Patients with LGE imbibitions also had significantly different eccentricity of the left ventricle, however in close numeric range to controls (without LGE imbibition). Correlations of LGE with the geometry of the left ventricle were significant, though mild, leading to an assumption that LGE is not in a straightforward relation with geometry, and that it is more likely secondarily changed due to infrastructural rearrangements of the left ventricle which consequently ended up as geometric eccentricity [31, 32]. In head to head settings, MaxMinEDDR was shown to be a superior diagnostic parameter than LGE, of similar diagnostic efficiency as the NC/C ratio or NC affecting more than 20% of the left ventricle, with the last two considered as among the best currently available evidence for diagnosing the LVNC [3, 12].

Geometry of the left ventricle was found to be connected with functional prognostic parameters such as systolic function or left atrial size [27]. The latter implies that local infrastructural alternations in terms of non-compaction eventually cause changes in the structure and function beyond the left ventricle, which could serve as a diagnostic landmark of the turning point of the failing heart. Systolic function of the left ventricle correlated with maximal and minimal end diastolic diameters in mid apical short axis slices, but not with MaxMinEDDR. This was further confirmed as a clinically insignificant diagnostic parameter of systolic dysfunction in ROC analysis. Based on those results, assumptions could be made that whilst systolic function of the left ventricle in patients with LVNC tends to be decreased, the initial disorder is probably hidden among the structural changes of non-compact myocardium, and eminently ends up with different grade of systolic dysfunction. There seems to be sequential relation, where systolic dysfunction lags in phase to infrastructural changes caused by non-compact myocardium and changes of geometry.

One must point out that these are preliminary investigations, focused on diagnostic utility of a single imaging modality scrutinized according to guidelines-based diagnostic criteria that are not perfect. The study was performed in the only large-volume tertiary center specialized for CMR in our country, on a selected population of cases with cardiovascular diseases and previous cardiovascular diagnostic and/or therapeutic workup. Retrospective study settings and selection of controls also might conceal limitations. Prospective validation, using multicentric settings, with inclusion of further clinical parameters, such as patients’ comorbidities and follow up of major adverse event rates is necessary to increase reproducibility.

## Conclusion

*In conclusion*, late gadolinium enhancement was found to be frequently expressed in patients with LVCN. These imbibitions were of insufficiently defined power to be used as a specific diagnostic parameter, in the context of LVNC. On the other hand, LGE was found to be a relatively valuable diagnostic landmark of complex infrastructural and functional changes within the failing heart. It correlated only mildly with left ventricle geometry in dedicated regions, where the latter was found to be a very promising novel LVNC diagnostic parameter. Geometric eccentricity associated with LVNC had a fine grade of diagnostic utility to tell apart the cases with non-ischemic cardiomyopathies from those with structurally normal heart. Segmental eccentricity was also in correlation with infrastructural and functional changes that even stretched beyond mid-apical regions, i.e. to the whole left ventricle and both atria. Changes of left ventricle geometry in addition to tissue characterization by late gadolinium imbibition seem to have potential for further contribution to the diagnostic imaging of the failing heart.

## Abbreviations

%: Percentages
+LR: Positive likelihood ratio
95%CI: 95%-confidence intervals
ARVD: Arrhythmogenic right ventricle dysplasia
AUC: Area under the curve
CMR: Cardiac magnetic resonance imaging
Compact (cm): End diastolic thickness of solid myocardial layer
DB: dark blood preparation
DCM: Dilative cardiomyopathy
ED: End diastolic
EDD: End diastolic dimension
EDV (mL): End diastolic volume in milliliters
ESV (mL): End systolic volume
Gd: Gadolinium
HASTE: Half-Fourier Acquisition Single-shot Turbo spin Echo
HCM: Hypertrophic cardiomyopathy
ICT2HI: Intracavital T-2-weighted hyperintensity
IVS (cm): Interventricular septum thickness in 4-chamber view
LA & RA (cm2): Left and right atrial area in square centimeters in 4-chamber view
LGE: Late gadolinium enhancement
LR: Negative likelihood ratio
LVEDD (cm): left ventricle end diastolic dimension in 4-chamber view
LVEF (%): Left ventricle ejection fraction
LVEF < 50%: Impairment of left ventricle systolic function
LVNC: Left ventricular non compaction
MA: Mid to apical slices in 6-mm pile stack of short axes
Max: Maximal
MaxMinEDDR: Maximal over minimal end diastolic dimension ratio
Min: Minimal
MM (gram): Myocardial mass in end-diastole
N: Number
NC > 20% LV: Percentage of trabeculations over 20% in the total mass of the left ventricle
NC%LV: Left ventricle non-compaction proportion in the total mass of the left ventricle
NC:C: Non compact to compact myocardial layer thickness
NC:C: Non-compact to compact myocardial layer thickness ratio
Non-compact (cm): ED thickness of trabeculated part i.e. non-compaction myocardium
P: Statistical significance
Rho-CC: Spearman’s rho correlation coefficient
ROC: Receiver operating characteristic
RV (cm): Right ventricle end diastolic dimension in 4-chamber view
SD: Standard deviations
SSFP: Steady state free precession
STIR: Short tau inversion recovery
SV (mL): Stroke volume
T2: relaxation - progressive dephasing of spinning dipoles following the 90° pulse i.e. spin-spin relaxation
TSE: turbo spin echo
